# Circular RNA circPOFUT1 enhances malignant phenotypes and autophagy-associated chemoresistance via sequestrating miR-488-3p to activate the PLAG1-ATG12 axis in gastric cancer

**DOI:** 10.1038/s41419-022-05506-0

**Published:** 2023-01-09

**Authors:** Ming Luo, Xiaofeng Deng, Zonglin Chen, Yongjun Hu

**Affiliations:** Department of General Surgery, The Second Xiangya Hospital, Central South University, Changsha, 410011 Hunan Province China

**Keywords:** Gastric cancer, Gastric cancer

## Abstract

Circular RNAs are key regulators in regulating the progression and chemoresistance of gastric cancer (GC), suggesting circular RNAs as potential therapeutic targets for GC. The roles of a novel circular RNA circPOFUT1 in GC are unknown. Here, we found that circPOFUT1 was upregulated in GC tissues and cells, and increased circPOFUT1 expression indicated poor prognosis. Overexpression of circPOFUT1 enhanced cell proliferation, migration, invasion and autophagy-associated chemoresistance in GC, which were suppressed by miR-488-3p overexpression. CircPOFUT1 reduced miR-488-3p expression via sponging miR-488-3p in GC cells. PLAG1 interacted with ATG12 and promoted its expression. MiR-488-3p bound to PLAG1 and suppressed the expression of PLAG1 and ATG12 in GC cells. Overexpression of circPOFUT1 enhanced autophagy-associated chemoresistance of GC cells in vivo, but it was inhibited by overexpression of miR-488-3p. Collectively, circPOFUT1 directly sponged miR-488-3p to activate the expression of PLAG1 and ATG12, thus enhancing malignant phenotypes and autophagy-associated chemoresistance in GC. Our findings show the potential of circPOFUT1 as biomarkers and targeting circPOFUT1 as a therapeutic strategy for GC.

## Introduction

Gastric cancer (GC) is responsible for estimated 1,090,000 new cases and over 750,000 new deaths in 2020 globally [1]. Although the five-year survival rate for localized GC is 70%, the overall survival rate for all stages is as low as approximately 30%. Growing advances have been achieved in GC treatment including surgery, chemotherapy, radiotherapy, targeted therapy and immunotherapy [2]. However, the therapeutic outcome is unsatisfactory for patients with advanced GC. Hence, elucidating the mechanisms underlying GC cell malignancy is crucial for developing novel therapeutic strategies.

Cisplatin, also called platinum diamminodichloride (DDP), is a major chemotherapeutic drug for GC patients. Combination chemotherapy with cisplatin and other compounds such as docetaxel, 5-fluorouracil and oxaliplatin shows promising effects [3, 4]. However, many patients develop cisplatin chemoresistance causing poor therapeutic outcome. Uncovering molecular mechanisms underlying GC chemoresistance is important. Growing evidence has revealed that autophagy, a survival strategy of cancer cells for adapting to stresses, elicits pro-survival effects in response to chemotherapy and promotes chemoresistance [5, 6]. Moreover, chemotherapy-induced autophagy protects cancer cells from fetal cellular damage and is emerging as a mechanism of chemoresistance.

Circular RNAs (circRNAs) are a novel class of non-coding RNAs forming a closed loop through covalent linkage between 5ʹ and 3ʹ termini and have emerged as key regulators in various pathophysiological processes including cancers [7, 8]. Hong and colleagues proved that circCRIM1 was upregulated in nasopharyngeal carcinoma (NPC) and promoted NPC chemoresistance via upregulating FOXQ1 [9]. Apart from being an important regulator in cancers, circRNAs have many other beneficial features such as tissue-specific expression, stable structure and high abundance in body fluids making circRNAs as promising biomarkers for cancers [10, 11]. Protein O-fucosyltransferase 1 (POFUT1) is highly expressed in GC, and its expression is positively correlated with aggressive tumor phenotypes, such as higher T and N classification as well as poor differentiation, indicating POFUT1 may facilitates its development as a biomarker for prognosis of GC [12]. Intriguingly, we identified a novel circular RNA circPOFUT1 encoded by *POFUT1*. Importantly, the function of circPOFUT1 in GC is unknown.

Many circRNAs function as competing endogenous RNAs (ceRNAs) to sponge microRNAs (miRNAs) and subsequently upregulate downstream genes, thereby regulating the progression and chemoresistance of various cancers [13, 14]. Zhu et al. reported that circNHSL1 promoted GC progression by functioning as a miR-1306-3p sponge to relieve its repressive effect on downstream target SIX1 [15]. Therefore, we hypothesized that circPOFUT1 might regulate the malignancy and chemoresistance in GC via acting as a ceRNA to sponge miRNAs and promote the expression of downstream target genes. Indeed, we demonstrated that circPOFUT1 directly sponged miR-488-3p to upregulate PLAG1 and ATG12, thereby promoting GC progression and cisplatin chemoresistance. Our study identifies a novel circRNA and reveals its roles in regulating GC progression and autophagy-associated chemoresistance, suggesting potential biomarkers and therapeutic targets for GC.

## Materials and methods

### Clinical specimens

Tumor and adjacent normal tissues were surgically resected and collected from 46 patients diagnosed with GC at the Second Xiangya Hospital, Central South University, which were snap-frozen for subsequent analysis of circPOFUT1 and miR-488-3p. Patient survival was monitored for 60 months. Patients were informed and provided written informed consent. Our study got approval from the Ethics Committee of the Second Xiangya Hospital, Central South University.

### Cell culture and treatment

Normal human epithelial GES-1 cells and GC cells BGC-823, AGS, HGC-27, MGC-803, SGC-7901 and MKN-45 were provided by the Cell Bank of Chinese Academy of Sciences (Shanghai, China) and cultured in RPMI 1640/10% fetal bovine serum (ThermoFisher Scientific, Waltham, MA, USA). For actinomycin D treatment, SGC-7901 cells were treated with actinomycin D from Sigma (St. Louis, MO, USA) at 5 µg/mL for 0, 4, 8, 12 and 24 hours. Subsequently, RNA was extracted for assessing the stability of circPOFUT1 and POFUT1 mRNA. For cisplatin (DDP) treatment, cells were treated with DDP (Sigma) at 2 µg/mL for 12 h [16].

### Cell transfection

CircPOFUT1 was cloned into the pcDNA3.1(+) CircRNA Mini Vector (OE-circPOFUT1, Addgene, Watertown, MA, USA) for overexpression. The coding regions of ATG12 and PLAG1 were inserted into the pcDNA 3.1 vector (pcDNA3.1-ATG12 and pcDNA3.1-PLAG1, ThermoFisher Scientific) for overexpression. Empty vectors (OE-NC and Vector) were used as negative controls. ShRNAs against circPOFUT1 (sh-circPOFUT1#1 and #2) and ATG12 (sh-ATG12), miR-488-3p mimics, locked nucleic acid (LNA)-miR-488-3p inhibitor, the siRNA against PLAG1 (si-PLAG1) and negative controls (sh-NC, si-NC, miR-NC and LNA-NC) were purchased from RiboBio (Guangzhou, China). SGC-7901, MKN-45 and SGC-7901/DDP cells were transfected with following combinations of constructs: sh-NC, sh-circPOFUT1, OE-NC, OE-circPOFUT1, miR-488-3p mimics + OE-NC, miR-488-3p mimics + OE-circPOFUT1, LNA-NC, LNA-miR-488-3p inhibitor, OE-circPOFUT1 + sh-ATG12, sh-circPOFUT1 + LNA-miR-488-3p inhibitor, sh-circPOFUT1 + pcDNA3.1-ATG12, si-NC, si-PLAG1, vector, pcDNA3.1-PLAG1, miR-NC, miR-488-3p mimics, miR-NC + vector, miR-488-3p mimics + vector, miR-488-3p + pcDNA3.1-PLAG1, LNA-NC + si-NC, LNA-miR-488-3p inhibitor + si-NC, LNA-miR-488-3p inhibitor + si-PLAG1 using the Lipo 3000 transfection reagent (ThermoFisher Scientific). For establishing stable cells with knockdown of circPOFUT1 and overexpression of miR-488-3p, sh-circPOFUT1 or miR-488-3p mimics were inserted into the pLKO.1 lentiviral vector, and lentiviral particles were packaged. Cells were then harvested for subsequent assays.

### In situ hybridization (ISH)

CircPOFUT1 expression in normal and GC tissues was examined via ISH. Briefly, tissues were embedded in paraffin and cut into 5-µm sections. Sections were then deparaffinized and incubated with biotin-conjugated circPOFUT1A probes overnight at 40 °C. Next day, sections were incubated with streptavidin-HRP (ThermoFisher Scientific), and DAB substrate (Beyotime, Shanghai, China) was added to detect circPOFUT1 followed by hematoxylin staining.

### Fluorescence in situ hybridization (FISH)

FISH was conducted as previously described with modification [17]. Briefly, SGC-7901 and MKN-45 cells were seeded on coverslips and grown to 70-80% confluency, which were rinsed and fixed in methanol/acetic acid (v/v, 3:1) solution for 30 minutes. Excess liquid was removed, and coverslips were air-dried followed by pre-dehydration in gradient ethanol solutions (70%, 80% and 95%). The Cy3-labeled circPOFUT1 probe was denatured at 80 °C for 10 min in hybridization solution. Coverslips were denatured at 72 °C for 5 min and re-dehydrated in ethanol solutions and air-dried. Subsequently, coverslips were incubated in hybridization solution containing the Cy3-labeled circPOFUT1 probe (30 nM, RiboBio) overnight at 37 °C. Subsequently, coverslips were rinsed and stained with DAPI (Beyotime) for 10 min. Coverslips were mounted and imaged with an EVOS fluorescence microscope (ThermoFishr Scientific).

### Cell proliferation analysis

For CCK-8 assays, 5 × 10^3^ GC cells with indicated transfections were seeded and maintained for 1, 3, 5 or 7 days in 96-well plates. Culture medium was replaced with 100 µL of fresh medium, and CCK-8 (10 µL, Sigma) was added. Subsequently, cells were incubated for 5 hours, and OD 450 was recorded. For EdU incorporation, GC cells were seeded and cultured to 70% confluency. EdU was supplemented added into culture medium at 10 µM, and cells were incubated for 18 h. Then, cells were fixed and permeabilized. The reaction cocktail was prepared, and the Click-iT reaction was performed following the manual (ThermoFisher Scientific). Finally, DAPI was used for nuclear staining, and cells were imaged with an EVOS fluorescence microscope (ThermoFishr Scientific). For colony formation assays, 1 × 10^3^ GC cells with indicated transfections were seeded and cultured for 14 days in 6-well plates. Subsequently, cell colonies were fixed, stained in 0.1% crystal violet solution (Selleck, Houston, TX, USA) and imaged with the Olympus BX51 microscope (Tokyo, Japan).

### Transwell assays for analyzing cell migration and invasion

GC cells with indicated transfections were seeded in the upper chamber. For analyzing cell invasion, the upper chamber was pre-coated with Matrigel (BD Biosciences, Franklin Lakes, NJ, USA), and cells were seeded in the upper chamber. After 24 hours, cells which migrated or invaded into the lower chamber were fixed, stained in crystal violet solution (Selleck) and imaged with the Olympus BX51 microscope. Transwell chambers were bought from Corning (Corning, NY, USA).

### MTT assays for determining IC50 value of DDP

SGC-7901 and SGC-7901/DDP cells were treated with DDP at 1, 2, 5, 10, 15 and 20 µg/mL for 12 h in 96-well plates. Cell viability was determined by MTT assays. In brief, the culture medium was replaced with 100 µL of fresh medium. MTT reagents (10 µL) were added and mixed gently. Cells were incubated for 4 h at 37 °C. SDS-Hcl solution (100 µL) was added, and microplates were incubated for additional 6 h. The absorbance at 450 nm was recorded. The concentrations of DDP which reduced cell viability to 50% were identified as IC50.

### Cell apoptosis

SGC-7901 and SGC-7901/DDP cells were transfected as indicated and treated with DDP. Cells were trypsinized detached and incubated in Annexin V binding buffer (100 µL) containing Annexin V-FITC and PI for 20 min. Subsequently, Annexin V binding buffer (400 µL) was added, and cell apoptosis was analyzed with BD FACSCalibur (BD Biosciences) in 30 min.

### Autophagy examination

For immunofluorescence (IF) staining of LC3, cells were washed, fixed and permeabilized. After wash, cells were blocked and incubated with a rabbit anti-LC3 antibody overnight. Next day, cells were rinsed and incubated with an Alexa Fluor 555-conjugated goat anti-rabbit secondary antibody (ThermoFisher Scientific) for 1 h. DAPI (Beyotime) was used for nuclear staining. Cells were imaged with an EVOS fluorescence microscope (ThermoFishr Scientific). For transmission electron microscope (TEM) observation of autophagosomes, cell pellets were rinsed and fixed in 2% glutaraldehyde solution for 1 h and in 1% OsO_4_ for 1 h. Subsequently, pellets were embedded in araldite, and ultra-thin sections were prepared and stained with uranyl acetate and lead citrate for imaging.

### Real-time quantitative reverse-transcription PCR (RT-qPCR)

Total RNA was isolated from GC tissues and cells using TRIzol™ LS Reagent (ThermoFisher Scientific) followed by electrophoresis for examining RNA quality. After quantification, RNA was reversely transcribed into cDNA using the SuperScript IV First-Strand Synthesis System (ThermoFisher Scientific). MiRNAs were extracted using the mirPremier microRNA Isolation Kit (Merck, St. Louis, MO, USA) and reversely transcribed into cDNA with the miScript kit from QIAGEN (Germantown, MD, USA). The expression of circPOFUT1, POFUT1, miR-488-3p, PLAG1 and ATG12 were examined by quantitative PCR. GAPDH and U6 snRNA were used as normalized controls. 2^−∆∆Ct^ method was used for calculation. Primers were as followed, circPOFUT1-F, 5ʹ-CCGTACCTTGGCTGTCCCTC-3ʹ, circPOFUT1-R, 5ʹ-TAGCAGCTTTGCAAATGCCAGA-3ʹ; POFUT1-F, 5ʹ-AACAGCTCTTCAAAGGGAAG-3ʹ, POFUT1-R, 5ʹ-ACAGTTGCCAATAAAGTGGT-3ʹ; miR-488-3p-F, 5ʹ-CGGGGCAGCUCAGUACAG-3ʹ, miR-488-3p-R, 5ʹ-CAGTGCGTGTCGTGGAGT-3ʹ; PLAG1-F, 5ʹ-GAGGGAGGATGTTAAAGCCC-3ʹ, PLAG1-R, 5ʹ-GCTCCAAACTCTAGCAAGGC-3ʹ; ATG12-F, 5ʹ-TTGTGGCCTCAGAACAGTTG-3ʹ, ATG12-R, 5ʹ-GAGAGTTCCAACTTCTTGGTCTG-3ʹ; U6 snRNA-F, 5ʹ-CTCGCTTCGGCAGCACATATACTA-3ʹ, U6 snRNA-R, 5ʹ-ACGAATTTGCGTGTCATCCTTGCG-3ʹ; GAPDH-F, 5ʹ-GTCTCCTCTGACTTCAACAGCG-3ʹ, GAPDH-R, 5ʹ-ACCACCCTGTTGCTGTAGCCAA-3ʹ.

### Western blotting

Cells were lysed in RIPA lysis buffer, and cell lysates were harvested. Protein (20 µg) was loaded for electrophoresis and subsequently transferred onto PVDF membranes (GE healthcare, Chicago, IL, USA). Membranes were blocked in 8% non-fat milk solution and incubated with rabbit anti-LC3 (1:2000, Abcam, Cambridge, UK), anti-Beclin1 (1:1000, Abcam), anti-p62 (1:1000, Abcam), anti-ATG12 (1:2000, Abcam) and anti-GAPDH (1:8000, Abcam). Subsequently, membranes were incubated with an HRP-conjugated goat anti-rabbit IgG antibody (Abcam) for 1 h. The bands were visualized using the ECL substrate (Beyotime). Image J software was used to analyze band intensity.

### RNA pull-down assays

For RNA pull-down, cells were lysed, and lysates were collected after centrifugation. The biotin-labeled circPOFUT1 probe was added into cell lysates and samples were incubated for 6 h at 4 °C. Subsequently, streptavidin-conjugated magnetic beads were added and incubated for 2 h with gentle rotation. Then, RNA was recovered, and the enrichment of circPOFUT1, hsa-miR-1234, hsa-miR-1245, hsa-miR-488-3p, hsa-miR-503, hsa-miR-518a-5p, hsa-miR-527, hsa-miR-646 and hsa-miR-888 was analyzed by RT-qPCR. The magnetic RNA-Protein pull-down kit was purchased from ThermoFisher Scientific.

### Dual-luciferase assays

Wildtype and mutant binding sites for miR-488-3p in circPOFUT1 (circPOFUT1-MT and circPOFUT1-MUT) and the 3ʹ untranslated region (UTR) of PLAG1 (PLAG1-WT and PLAG1-MUT) were cloned into the pmirGLO vector (Promega, Madison, WI, USA). Promoter regions of different lengths (~2000bp and 500 bp) were cloned into the pGL3 vector (Promega). SGC-7901 cells were co-transfected with following combinations: circPOFUT1 reporters + miR-488-3p mimics, PLAG1 reporters + miR-488-3p mimics, ATG12 promoter reporters + pcDNA3.1-PLAG1 or ATG12 promoter reporters + si-PLAG1. NC mimics, vector and si-NC were used as negative controls. After 48 h, cells were harvested, and the luciferase activity was examined using the Dual-Glo system (Promega).

### Chromatin immunoprecipitation (ChIP)

ChIP assays were performed using the magnetic ChIP kit (ThermoFisher Scientific). Briefly, SGC-7901 cells (~4 × 10^6^ cells) were cross-linked in 1% formaldehyde solution. Glycine solution was added and incubated for 5 min, and cells were detached by scraping. Cells were centrifugated and the supernatants were removed. Cell pellets were resuspended in Membrane Extraction Buffer supplemented with protease and phosphatase inhibitors and incubated for 10 min on ice. After centrifugation, nuclei were digested in MNase for 15 min at 37 °C. Subsequently, nuclei were sonicated to obtain DNA-protein fragments. The fragments were subjected to immunoprecipitation with a ChIP-grade rabbit anti-PLAG1 or normal rabbit IgG. Protein A/G Magnetic Beads were mixed with the immunoprecipitated complex and incubated with gentle rotation. DNA was eluted and recovered for RT-qPCR analysis.

### A subcutaneous GC xenograft mouse model

Athymic nude mice (female, 4-week-old) were obtained from the Ethics Committee of the Second Xiangya Hospital, Central South University. All animal procedures complied with National Institutes of Health guidelines. The Animal Care and Use Committee of the Ethics Committee of the Second Xiangya Hospital, Central South University approved our study. The subcutaneous GC xenograft mouse model was established as previously reported [18]. SGC-7901 and SGC-7901/DDP cells which were stably transfected as indicated were subcutaneously injected into the left flanks of mice (1 × 10^7^ cells each mouse, *n* = 5 per group). DDP was intraperitoneally injected into mice at 10 mg/kg once a week. Tumor size was measured at 0, 7, 14, 21 and 28 days, and tumor volumes were calculated with the formula length × width^2^/2. Finally, tumors were resected and weighed.

### Immunohistochemistry (IHC) staining

Subcutaneous tumors were excised, embedded and cut into 5-µm sections. Sections were deparaffinized, rehydrated and subjected to antigen retrieval in citrate buffer (pH 6.0) for 15 min at 95 °C. Sections were immersed in 0.3% H_2_O_2_ solution and blocked in 10% BSA solution. Sections were incubated with a rabbit ATG12 (1:50, Abcam) or Ki-67 (1:100, Abcam) antibody for 3 h. Sections were washed and probed with an HRP-conjugated goat anti-rabbit antibody for 1 hour. DAB (Beyotime) was used for visualizing the signal. Sections were stained with hematoxylin (Beyotime), mounted and imaged the Olympus BX51 microscope.

### Statistical analysis

Data was from three independent experiments and expressed as mean ± standard deviation. The Student’s *t* test and one-way analysis of variance (ANOVA) were applied to compare the variance of two and multiple groups, respectively. The Kaplan-Meier plotter was applied to assess the variance of survival between patients with high and low expression of circPOFUT1. The correlation between the expression of circPOFUT1 and miR-488-3p in GC tissues was evaluated using the Spearman’s correlation. *P* < 0.05 was statistically significant.

## Results

### CircPOFUT1 was highly expressed in GC tissues and cells

**Fig. 1:**
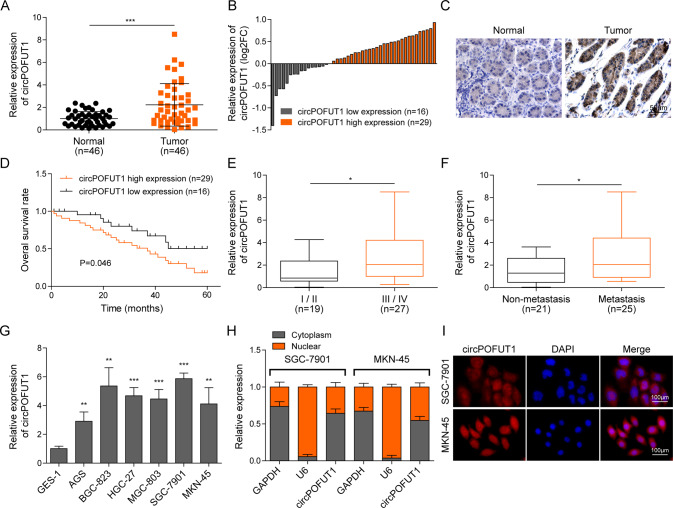
CircPOFUT1 was highly expressed in GC tissues and cells. **A** RT-qPCR analysis of circPOFUT1 (*n* = 46). **B** GC patients were divided into two groups, low- (*n* = 16) and high-expression (*n* = 30) groups. **C** CircPOFUT1 expression in normal and GC tissues was analyzed by ISH (scale bar, 50 µm). **D** The survival of patients. **E** The expression of circPOFUT1 in tumors at stage I/II (*n* = 19) and III/IV (*n* = 27). **F** The expression of circPOFUT1 in tumors from patients with non-metastatic (*n* = 21) and metastatic cancers (*n* = 25). **G** RT-qPCR analysis of circPOFUT1 (*n* = 3). **H** RT-qPCR analysis of circPOFUT1 in cytoplasmic and nuclear fractions (*n* = 3). **I** The localization of circPOFUT1 (Red) was validated by RNA-FISH staining (scale bar, 100 µm; Nuclei, Blue). *P < 0.05, **P < 0.01, and ***P < 0.001.

Tumor and adjacent normal tissues were collected from GC patients, and GC tissues showed elevated circPOFUT1 expression (Fig. 1A). Patients were divided into two groups, circPOFUT1 low and high expression groups (Fig. 1B). ISH assays showed increased expression of circPOFUT1 in GC tissues (Fig. 1C). The survival of patients with high circPOFUT1 expression was reduced (Fig. 1D). We observed higher circPOFUT1 expression in patients at advanced stages (stage III/IV) and patients with metastasis (Fig. 1E, F). GC cells including AGS, BGC-823, HGC-27, MGC-803, SGC-7901 and MKN-45 showed increased circPOFUT1 expression (Fig. 1G). CircPOFUT1 could be detected in both nuclear and cytoplasmic fractions, but it mainly localized in the cytoplasmic fraction (Fig. 1H, I).

### CircPOFUT1 characterization

**Fig. 2:**
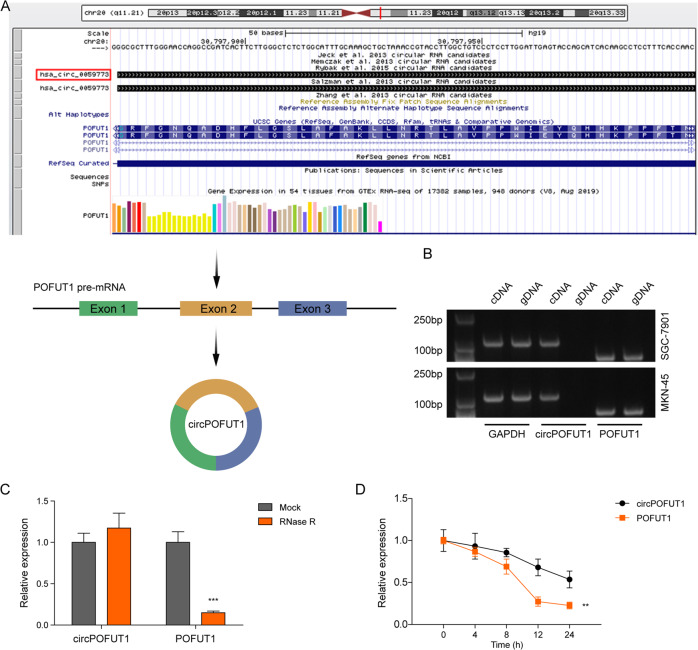
CircPOFUT1 characterization. **A** The schematic diagram illustrated the origination of circPOFUT1 from the exon2 of POFUT1 pre-mRNA. **B** RT-PCR was applied to examine circPOFUT1 in cDNA and gDNA using divergent and convergent primers. **C** RT-qPCR analysis of circPOFUT1 and POFUT1 mRNA in response to RNase R treatment (*n* = 3). **D** The stability of circPOFUT1 and POFUT1 in response to actinomycin D (*n* = 3). **P < 0.01 and ***P < 0.001.

CircPOFUT1 is located on chromosome q11.21 and originates from the exon 2 of POFUT1 pre-mRNA as illustrated in Fig. 2A. Both POFUT1 and GAPDH were amplified from complementary DNA (cDNA) and genomic DNA (gDNA), but circPOFUT1 amplification product was only observed in cDNA (Fig. 2B). CircPOFUT1, but not POFUT1, was resistant to RNase R treatment (Fig. 2C). Compared to liner POFUT1 mRNA, circPOFUT1 was stable with a half-life over 24 hours upon actinomycin D treatment (Fig. 2D). These observations confirmed circular structure and stable characteristics of circPOFUT1.

### CircPOFUT1 promoted GC cell proliferation, migration and invasion

**Fig. 3:**
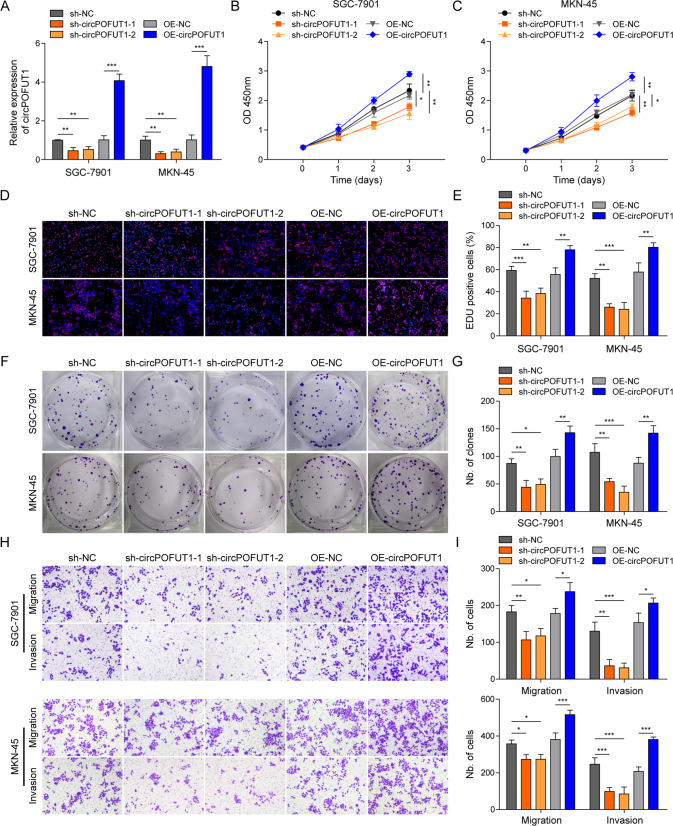
CircPOFUT1 promoted GC cell proliferation, migration and invasion. **A** RT-qPCR analysis of circPOFUT1 (*n* = 3). **B**, **C** Cell proliferation was analyzed by CCK-8 (*n* = 3). **D**, **E** EdU (Red) incorporation assays (*n* = 3; Nuclei, Blue). **F**, **G** Colony-forming capacity of GC cells (*n* = 3). **H**, **I** Transwell assays for migration and invasion (*n* = 3). *P < 0.05, **P < 0.01 and ***P < 0.001.

CircPOFUT1 was silenced or overexpressed in GC cells, which was confirmed by RT-qPCR (Fig. 3A). Neither circPOFUT1 knockdown nor overexpression affected POFUT1 expression (Supplementary Fig. 1A). CCK-8 assays showed that knockdown of circPOFUT1 restrained the proliferation of SGC-7901 and MKN-45 cells, but circPOFUT1 overexpression promoted cell proliferation (Fig. 3B, C). In addition, EdU incorporation was reduced by knockdown of circPOFUT1 but enhanced by circPOFUT1 overexpression (Fig. 3D, E). CircPOFUT1-silencing GC cells formed fewer colonies, but overexpression of circPOFUT1 enhanced colony formation (Fig. 3F, G). Furthermore, we found that migratory and invasive capacities were impaired by circPOFUT1 knockdown (Fig. 3H, I). Conversely, overexpression of circPOFUT1 promoted GC cell migration and invasion (Fig. 3H, I). Our findings suggested that circPOFUT1 promoted GC cell malignant phenotypes.

### CircPOFUT1 served as a sponge for miR-488-3p to promote GC malignancy

**Fig. 4:**
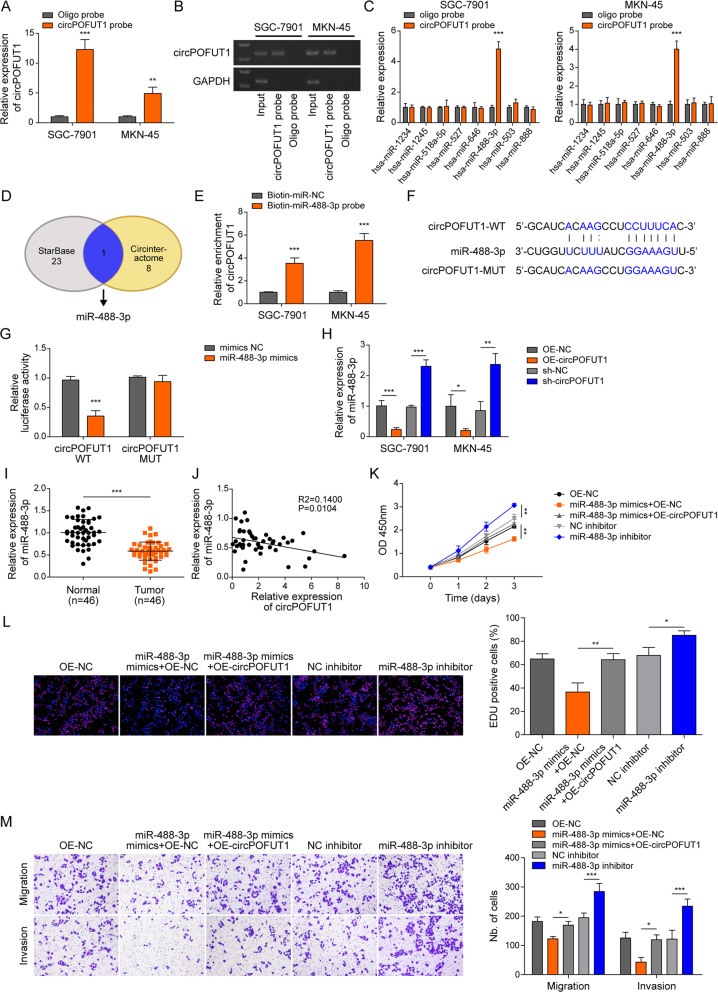
CircPOFUT1 served as a sponge for miR-488-3p to promote GC malignancy. **A**, **B** CircPOFUT1 was pulled down by the circPOFUT1 probe (*n* = 3). **C** The enrichment of hsa-miR-1234, hsa-miR-1245, hsa-miR-488-3p, hsa-miR-503, hsa-miR-518a-5p, hsa-miR-527, hsa-miR-646 and hsa-miR-888 by the circPOFUT1 probe was evaluated (*n* = 3). **D** Downstream miRNA targets of circPOFUT1 were predicted. **E** CircPOFUT1 was pulled down by the miR-488-3p probe (*n* = 3). **F** The wildtype and mutant binding sites for miR-488-3p in circPOFUT1. **G** The luciferase activity of wildtype (circPOFUT1-WT) and mutant (circPOFUT1-MUT) circPOFUT1 reporters (*n* = 3). **H** RT-qPCR analysis of miR-488-3p (*n* = 3). **I** The expression of miR-488-3p in GC and normal tissues (*n* = 46). **J** The correlation analysis of the expression of circPOFUT1 and miR-488-3p. **K** Cell proliferation was evaluated by CCK-8 (*n* = 3). **L** EdU (Red) incorporation assays (*n* = 3; Nuclei, Blue). **M** Transwell assays for migration and invasion (*n* = 3). *P < 0.05, **P < 0.01 and ***P < 0.001.

As circRNAs function as miRNA sponges to exert functions in human cancers [19], we determined to explore downstream miRNA targets of circPOFUT1 through RNA pull-down. CircPOFUT1 could be efficiently pulled down by the circPOFUT1 probe (Fig. 4A, B). Moreover, eight miRNAs were predicted to interact with circPOFUT1 through CircInteractome [20], and hsa-miR-488-3p was highly pulled down by the circPOFUT1 probe (Fig. 4C). However, hsa-miR-503 was slightly enriched, and other miRNAs including hsa-miR-1234, hsa-miR-1245, hsa-miR-518a-5p, hsa-miR-527, hsa-miR-646 and hsa-miR-888 could not be enriched by the circPOFUT1 probe (Fig. 4C). Furthermore, only miR-488-3p was overlapped in predicted downstream miRNAs of circPOFUT1 using starBase v2.0 [21] and CircInteractome (Fig. 4D). Therefore, we decided to focus the interaction of circPOFUT1 and miR-488-3p in this study. We found that circPOFUT1 could be efficiently pulled down by the miR-488-3p probe (Fig. 4E). A potential binding site for miR-448-3p in circPOFUT1 was predicted (Fig. 4F). The luciferase activity of the wildtype circPOFUT1 reporter, but not the mutant circPOFUT1 reporter, was reduced by miR-488-3p overexpression (Fig. 4G). Additionally, miR-488-3p was downregulated in circPOFUT1-overexpressing cells but upregulated by circPOFUT1 knockdown (Fig. 4H). MiR-488-3p was downregulated in GC tissues, and the expression of miR-488-3p and circPOFUT1 was negatively correlated (Fig. 4I, J). These results suggested that circPOFUT1 served as a miR-488-3p sponge to reduce its abundance in GC. Furthermore, miR-488-3p overexpression reduced SGC-7901 cell proliferation, whereas miR-488-3p inhibitor promoted proliferation (Fig. 4K, L). Intriguingly, simultaneous circPOFUT1 overexpression reversed miR-488-3p-mediated inhibition of cell proliferation (Fig. 4K, L). Also, the migration and invasion of SGC-7901 cells were suppressed by miR-488-3p overexpression, which was abolished by simultaneous circPOFUT1 overexpression (Fig. 4M). Knockdown of miR-488-3p promoted cell migration and invasion (Fig. 4M). Our data indicated that circPOFUT1 sponged miR-488-3p to promote GC malignancy.

### CircPOFUT1 facilitated autophagy-associated chemoresistance by targeting miR-488-3p

**Fig. 5:**
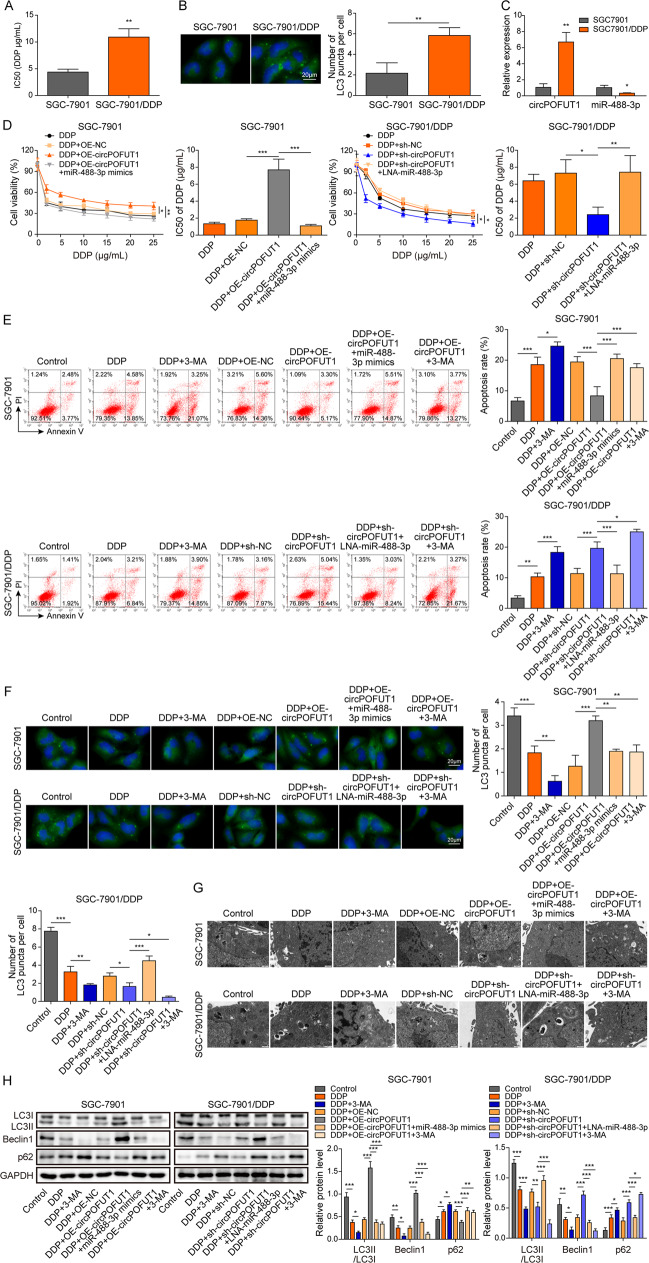
CircPOFUT1 facilitated autophagy-associated chemoresistance through targeting miR-488-3p. **A** The IC50 of DDP (*n* = 3). **B** IF staining of LC3 (Green; Scale bar, 20 µm; Nuclei, Blue). **C** The expression of circPOFUT1 and miR-488-3p (*n* = 3). **D** Cell viability analysis after DDP treatment (*n* = 3). **E** Cell apoptosis was examined by Annexin V and PI staining (*n* = 3). **F** IF staining of LC3 (Green; Scale bar, 20 µm; Nuclei, Blue). **G** Autophagosomes were observed by electron microscope. **H** Protein levels of LC3II/I, Beclin1 and p62 (*n* = 3). *P < 0.05, **P < 0.01 and ***P < 0.001.

We examined whether circPOFUT1 regulates cisplatin chemoresistance of GC cells. SGC-7901 and cisplatin-resistant SGC-7901 (SGC-7901/DDP) cells were treated with DDP. SGC-7901/DDP cells showed considerably higher IC50 than SGC-7901 cells, indicating its chemoresistance to DDP (Fig. 5A). Intriguingly, we observed raised LC3 dots in SGC-7901/DDP cells, suggesting that autophagy might be enhanced in cisplatin-resistant cells (Fig. 5B). Meanwhile, increased circPOFUT1 expression and reduced miR-488-3p expression were observed in SGC-7901/DDP cells (Fig. 5C). Overexpression of circPOFUT1 raised the IC50 of DDP in SGC-7901 cells, but this effect was abrogated by miR-488-3p overexpression (Fig. 5D). Knockdown of circPOFUT1 reduced the IC50 of DDP in SGC-7901/DDP cells, but this effect was abrogated by locked nucleic acids (LNA)-miR-488-3p (Fig. 5D). DDP caused notably enhanced SGC-7901 cell apoptosis, but the pro-apoptotic effect was moderate in SGC-7901/DDP cells, and 3-MA, an autophagy inhibitor, enhanced DDP-induced cell apoptosis (Fig. 5E). DDP-induced apoptosis was inhibited by overexpression of circPOFUT1, which was reversed by simultaneous miR-488-3p overexpression or 3-MA treatment (Fig. 5E). The moderate apoptosis in SGC-7901/DDP cells was further enhanced by knockdown of circPOFUT1 or 3-MA treatment, but it was abrogated by LNA-miR-488-3p, and 3-MA treatment further enhanced circPOFUT1 silencing-induced apoptosis (Fig. 5E). To explore the implication of circPOFUT1 in the regulation of autophagy, GC tissues were divided into LC3 dots ^More^ and LC3 dots ^Less^ groups based on the median number of LC3 dots (Supplementary Fig. 1B), and LC3 dots ^More^ GC tissues showed increased circPOFUT1 expression compared to LC3 dots ^Less^ GC tissues (Supplementary Fig. 1C), which was further confirmed by ISH (Supplementary Fig. 1D, E). Therefore, we focused whether circPOFUT1 regulated chemoresistance through autophagy. Furthermore, LC3 and autophagosomes were downregulated in SGC-7901 cells but moderately reduced in SGC-7901/DDP cells after DDP treatment, and 3-MA treatment further reduced LC3 puncta (Fig. 5F, G). Overexpression of circPOFUT1 enhanced LC3 expression and autophagosome formation in SGC-7901 cells, but it was inhibited by miR-488-3p mimics and 3-MA treatment (Fig. 5F, G). Moreover, knockdown of circPOFUT1 reduced LC3 expression and autophagosome formation in SGC-7901/DDP cells, which was reversed by LNA-miR-488-3p but further reduced by 3-MA (Fig. 5F, G). In SGC-7901 cells, DDP inhibited the expression of LC3II/I and Beclin1 and promoted p62 expression, and these effects were enhanced by 3-MA treatment (Fig. 5H). Overexpression of circPOFUT1 upregulated LC3II/I and Beclin1 but downregulated p62, and these effects were abolished by miR-488-3p mimics and 3-MA (Fig. 5H). SGC-7901/DDP cells showed increased ratio of LC3II/I and Beclin1 and decreased p62 expression (Fig. 5H). DDP treatment reduced the expression of LC3II/I and Beclin1 and upregulated p62, which was enhanced by 3-MA (Fig. 5H). Meanwhile, knockdown of circPOFUT1 further enhanced DDP-mediated regulation of the expression of LC3II/I, Beclin1 and p62, which was reversed by LNA-miR-488-3p but strengthened by 3-MA treatment (Fig. 5H). These data suggested that circPOFUT1 promoted autophagy-associated chemoresistance by targeting miR-488-3p in GC.

### ATG12 was a downstream effector of circPOFUT1 in regulating autophagy-associated chemoresistance

**Fig. 6:**
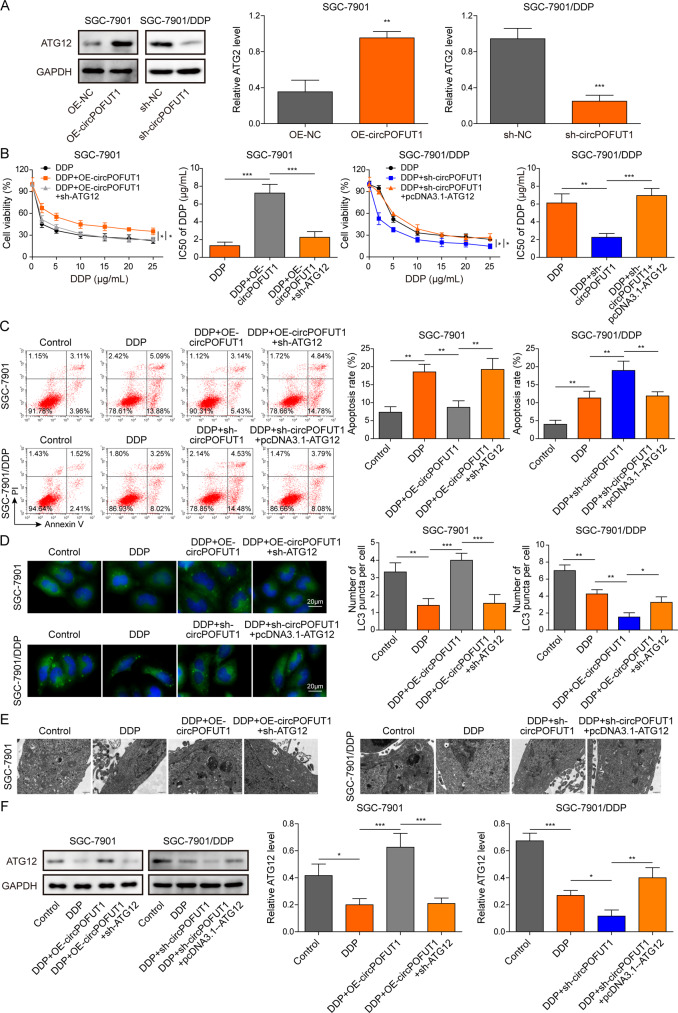
ATG12 was a downstream effector of circPOFUT1 in regulating autophagy-associated chemoresistance. **A** The expression of ATG12 was examined by western blotting (*n* = 3). **B** Cell viability analysis after DDP treatment (*n* = 3). **C** Cell apoptosis analysis (*n* = 3). **D** IF staining of LC3 (Green; Scale bar, 20 µm; Nuclei, Blue). **E** Autophagosomes were observed by electron microscope. **F** The expression of ATG12 was assessed by western blotting (*n* = 3). *P < 0.05, **P < 0.01 and ***P < 0.001.

Intriguingly, we found that ATG12, a key regulator in autophagy, was upregulated by overexpression of circPOFUT1 in SGC-7901 cells, and silencing of circPOFUT1 reduced its expression in SGC-7901/DDP cells (Fig. 6A). The IC50 of DDP in SGC-7901 cells was elevated by circPOFUT1 overexpression, but knockdown of ATG12 reduced the IC50 (Fig. 6B). Knockdown of circPOFUT1 reduced the IC50 of DDP in SGC-7901/DDP cells, but it was reversed by ATG12 overexpression (Fig. 6B). Furthermore, DDP-induced cell apoptosis was inhibited by overexpression of circPOFUT1, but this effect was abrogated by simultaneous ATG12 knockdown in SGC-7901 cells (Fig. 6C). In addition, knockdown of circPOFUT1 sensitized SGC-7901/DDP cells to cisplatin, which was reversed by overexpression of ATG12 (Fig. 6C). DDP-mediated reduction of the expression of LC3 and ATG12 and autophagosome formation was restored by circPOFUT1 overexpression in SGC-7901 cells, but knockdown of ATG12 reversed these effects (Fig. 6D–F). Knockdown of circPOFUT1 enhanced DDP-mediated moderate reduction of the expression of LC3 and ATG12 and autophagosomes in SGC-7901/DDP cells, but it was abrogated by simultaneous overexpression of ATG12 (Fig. 6D–F). We examined other ATGs and found that knockdown of circPOFUT1 showed strongest inhibitory effect on ATG12 expression (Supplementary Fig. 2A). These results implied that circPOFUT1 promoted ATG12 expression to regulate autophagy-associated chemoresistance in GC.

### PLAG1 functioned as a transcriptional activator of ATG12

**Fig. 7:**
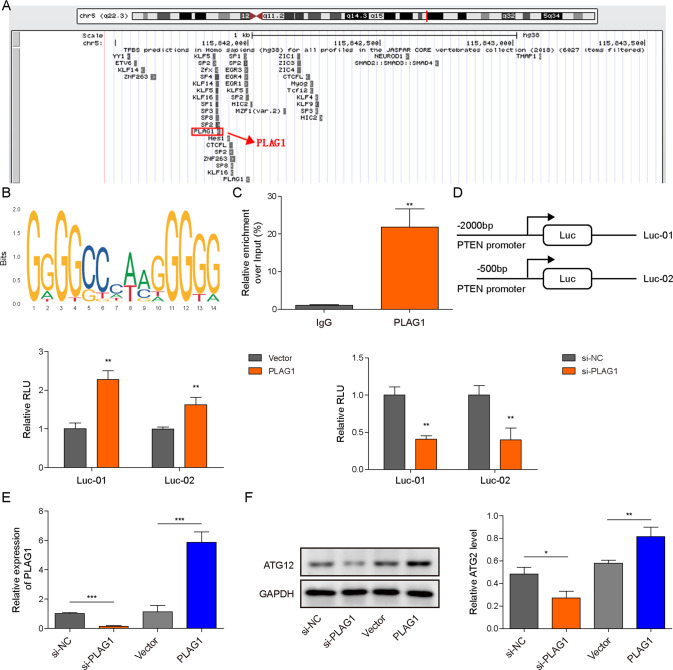
PLAG1 functioned as a transcriptional activator of ATG12. **A** The enrichment of PLAG1 on the promoter of ATG12 was identified using UCSC Genome Browser Home. **B** A binding site for PLAG1 in the promoter region of ATG12 was predicted using JASPAR database. **C** The binding of PLAG1 to the promoter of ATG12 was validated by ChIP assays (*n* = 3). **D** The luciferase activity of ATG12 reporters (*n* = 3). **E**, **F** The expression of PLAG1 and ATG12 (*n* = 3). *P < 0.05, **P < 0.01 and ***P < 0.001.

PLAG1, a well-known transcriptional activator [22], potentially bound to the promoter of ATG12 (Fig. 7A, B). Moreover, we found that miR-488-3p was expressed at a low level, but upregulation of PLAG1 and ATG12 was observed in stomach adenocarcinoma (STAD) from The Cancer Genome Atlas (TCGA) (Supplementary Fig. 2B), suggesting a potential regulatory relationship among them. ChIP assays showed significant enrichment of PLAG1 in the promoter of ATG12 (Fig. 7C). The luciferase activity of ATG12 reporters was obviously enhanced by overexpression of PLAG1 but greatly reduced by PLAG1 silencing (Fig. 7D). Furthermore, overexpression of PLAG1 promoted ATG12 expression, whereas knockdown of PLAG1 reduced ATG12 expression (Fig. 7E, F). Collectively, our findings demonstrated that PLAG1 directly targeted ATG12 as a transcriptional activator to promote its expression in GC.

### MiR-488-3p directly targeted PLAG1 to reduce ATG12 expression

**Fig. 8:**
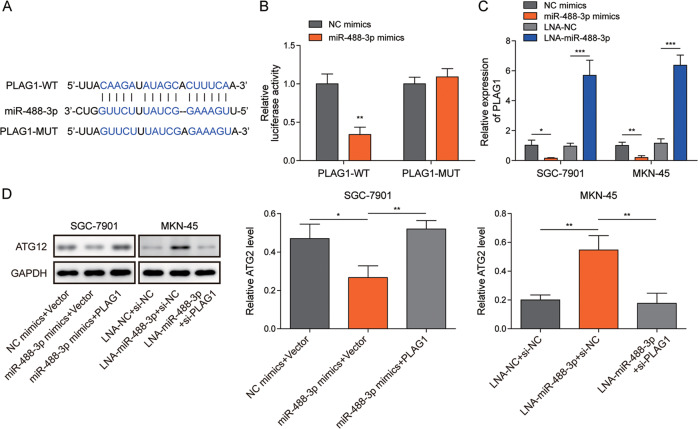
MiR-488-3p directly targeted PLAG1 to negatively regulate ATG12 expression in GC. **A** The wildtype and mutant binding sites for miR-488-3p in the 3ʹUTR of PLAG1. **B** The luciferase activity of wildtype (PLAG1-WT) and mutant (PLAG1-MUT) PLAG1 reporters (*n* = 3). **C** RT-qPCR analysis of PLAG1 (*n* = 3). **D** The expression of ATG12 was assessed by western blotting (*n* = 3). *P < 0.05, **P < 0.01 and ***P < 0.001.

MiRNAs can bind to downstream targets to reduce their expression, but no binding sites for miR-488-3p in ATG12 was identified by bioinformatics, suggesting that miR-488-3p might regulate ATG12 indirectly. Interestingly, a potential binding site for miR-488-3p in the 3ʹ UTR of PLAG1 was predicted through bioinformatics (Fig. 8A). The luciferase activity of the wildtype PLAG1 reporter (PLAG1-WT) was significantly reduced by overexpression of miR-488-3p (Fig. 8B). Moreover, overexpression of miR-488-3p inhibited PLAG1 expression, whereas knockdown of miR-488-3p significantly enhanced PLAG1 expression (Fig. 8C). In addition, ATG12 was downregulated by overexpression of miR-488-3p, which was reversed by PLAG1 overexpression (Fig. 8D). Knockdown of miR-488-3p enhanced ATG12 expression, and it was suppressed by simultaneous silencing of PLAG1 in MKN-45 cells (Fig. 8D). These findings suggested that miR-488-3p directly bound to PLAG1 to reduce its expression, thus inhibiting ATG12 expression in GC.

### CircPOFUT1 accelerated tumor growth via targeting miR-488-3p to promote autophagy-associated chemoresistance in vivo

A subcutaneous GC xenograft mouse model was established. DDP treatment greatly reduced tumor size and weight (Supplementary Fig. 3A–C). DDP-mediated suppression of tumor growth was abrogated by circPOFUT1 overexpression, whereas simultaneous overexpression of miR-488-3p re-sensitized GC cells to DDP (Supplementary Fig. 3A–C). Additionally, DDP treatment moderately suppressed tumor growth of SGC-7901/DDP cells (Supplementary Fig. 3D–F). Knockdown of circPOFUT1 further reduced tumor size and weight, but it was counteracted by simultaneous miR-488-3p knockdown (Supplementary Fig. 3D–F). IHC staining showed that the expression of ATG12 and Ki-67 were reduced by DDP (Supplementary Fig. 3G). Overexpression of circPOFUT1 restored their expression, but overexpression of miR-488-3p inhibited the expression of ATG12 and Ki-67 (Supplementary Fig. 3G). In tumors formed by SGC-7901/DDP cells, knockdown of circPOFUT1 markedly reduced the expression of ATG12 and Ki-67, but it was reversed by miR-488-3p knockdown (Supplementary Fig. 3G). Our findings demonstrated that circPOFUT1 enhanced autophagy-associated chemoresistance via targeting miR-488-3p, thus promoting tumor growth.

## Discussion

Surgical resection is the first choice for GC patients at the early stage. However, over 60% of GC patients are firstly diagnosed with regional or distant metastasis [23]. Chemotherapy is the major option for these patients [24]. However, chemoresistance is a major cause of the poor outcome of GC patients [25, 26]. Autophagy serves key roles in regulating chemoresistance in GC. Emerging evidence has demonstrated that inhibition of autophagy can reduce chemoresistance and sensitize GC cells to cisplatin. Here, we firstly reported that a novel circular RNA circPOFUT1 was highly expressed in GC cells and circPOFUT1 enhanced autophagy-associated chemoresistance to cisplatin in GC. Our findings identify ciecPOFUT1 as a potential therapeutic target to sensitize GC cells to cisplatin.

Many circRNAs are abundant in various human cancers and have been proved to exert vital roles in cancer progression [27–29]. Most circRNAs are products that are spliced from pre-mRNAs [30]. In 2016, a study revealed POFUT1 expression in GC and its association with GC cell carcinoma and aggressiveness [12]. Through bioinformatics, we found a novel circRNA circPOFUT1 (hsa_circ_0059773) derived from the exon 2 of POFUT1 pre-mRNA, but circPOFUT1 expression and its roles in GC have not been reported. Here, we confirmed circular characteristics of circPOFUT1 and elevated circPOFUT1 expression in GC tissues. Overexpression of circPOFUT1 induced cell proliferation, migration and invasion and enhanced autophagy-associated chemoresistance, supporting that circPOFUT1 acted as an oncogene in GC.

CircRNAs sponge or sequester miRNAs to relieve miRNA-mediated suppression of target mRNAs [31, 32]. CircCACTIN was reported to promote GC growth and EMT via sponging miR-331-3p to enhance TGFBR1 expression [33]. We identified miR-488-3p as a target miRNA of circPOFUT1, and circPOFUT1 sponged miR-488-3p to reduce its abundance in GC. MiR-488-3p works as a tumor suppressor in many cancers including GC [34, 35] and sensitizes melanoma cells to cisplatin [36]. In consistence, we found that circPOFUT1 sponged miR-488-3p to promote GC cell malignancy and chemoresistance to cisplatin. Overexpression of miR-488-3p reversed circPOFUT1-mediated effects and promoted cell proliferation, migration and invasion, supporting the notion that miR-488-3p is a tumor suppressor in GC.

Autophagy-related proteins are key regulators in autophagy, among which ATG12 regulates autophagosome formation [37]. ATG12 is implicated in the regulation of chemoresistance via modulating autophagy in various cancers [18, 38, 39]. We observed abnormal autophagy and ATG12 expression in cisplatin-resistance GC cells, indicating the correlation between ATG12 and autophagy-associated chemoresistance. We did not find any evidence suggesting the direct interaction between miR-488-3p and ATG12, whereas a binding site for PLAG1 was identified in the promoter of ATG12. We confirmed that PLAG1 bound to the promoter of ATG12 and induced its expression in GC. We further validated that miR-488-3p bound to PLAG1 to reduce ATG12 expression, thus regulating chemoresistance and GC progression.

Taken together, we firstly demonstrated that circPOFUT1 accelerated GC progression and enhanced autophagy-associated chemoresistance to cisplatin by sponging miR-488-3p to upregulate PLAG1 and ATG12(Supplementary Fig. 4). Our works not only identifies a novel circRNA in GC and its roles in GC progression and chemoresistance, but also provides potential biomarkers and therapeutic targets for GC. For potential clinical application, more patient samples and in vivo assays should be adopted in future studies.

## Supplementary information

Original Data File

Supplementary Materials

aj-checklist

## Data Availability

The datasets used or analyzed during the current study are available from the corresponding author on reasonable request.
