# Supplementary figures and images for: Circular RNA circPOFUT1 enhances malignant phenotypes and autophagy-associated chemoresistance via sequestrating miR-488-3p to activate the PLAG1-ATG12 axis in gastric cancer
Source: Cell Death Dis. 2023 Jan 9;14(1):10. doi: 10.1038/s41419-022-05506-0 (PMC9829716; doi:10.1038/s41419-022-05506-0)

Supplemental Material

The sum of Original Western blot image

Fig5H


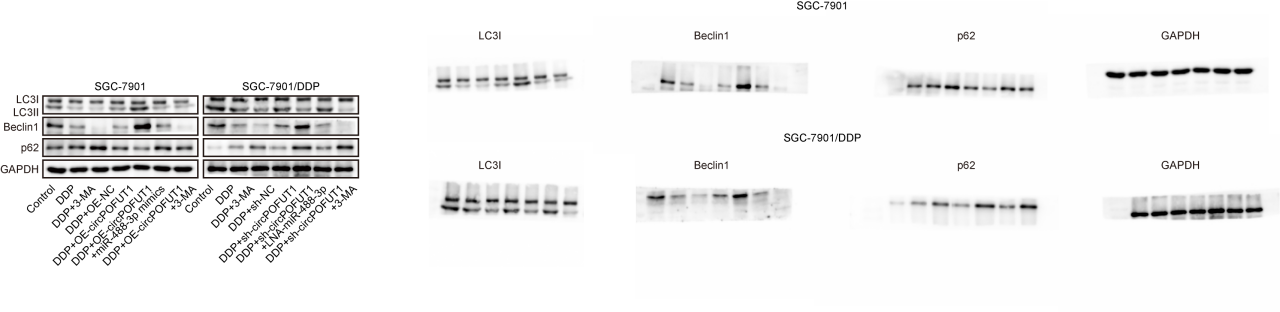


Fig6A


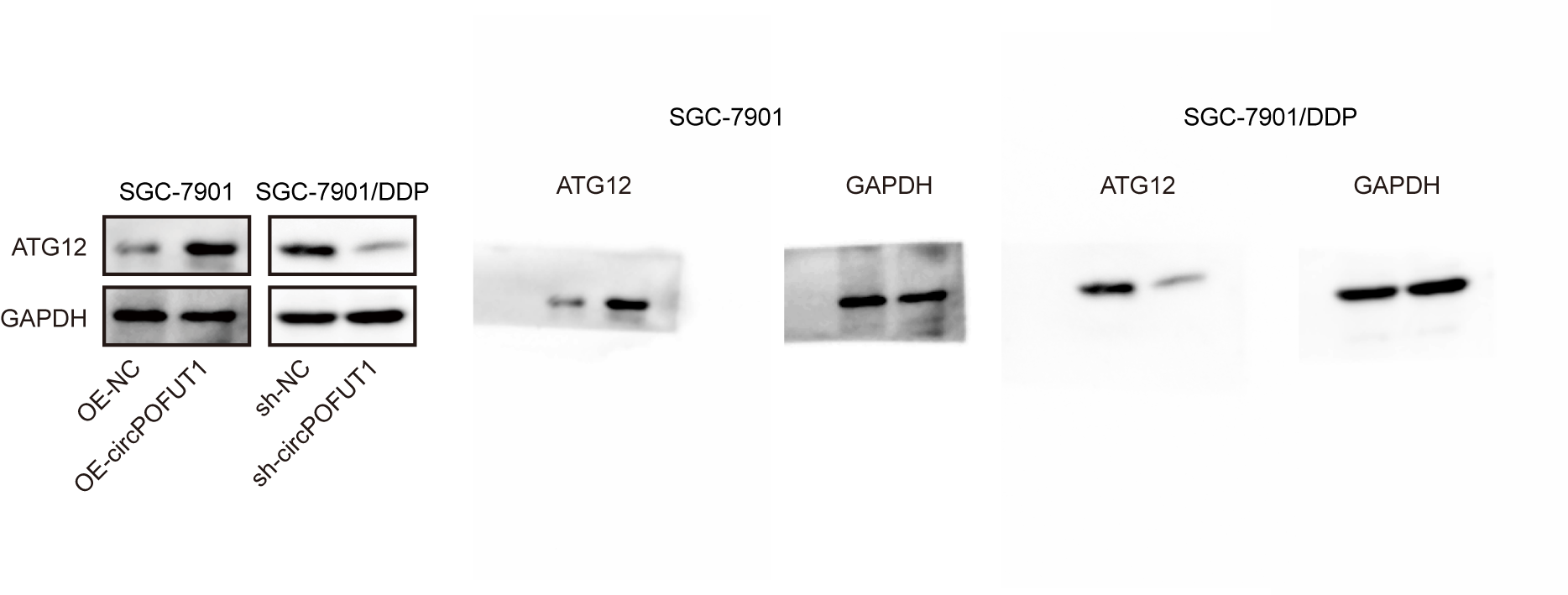


Fig6F


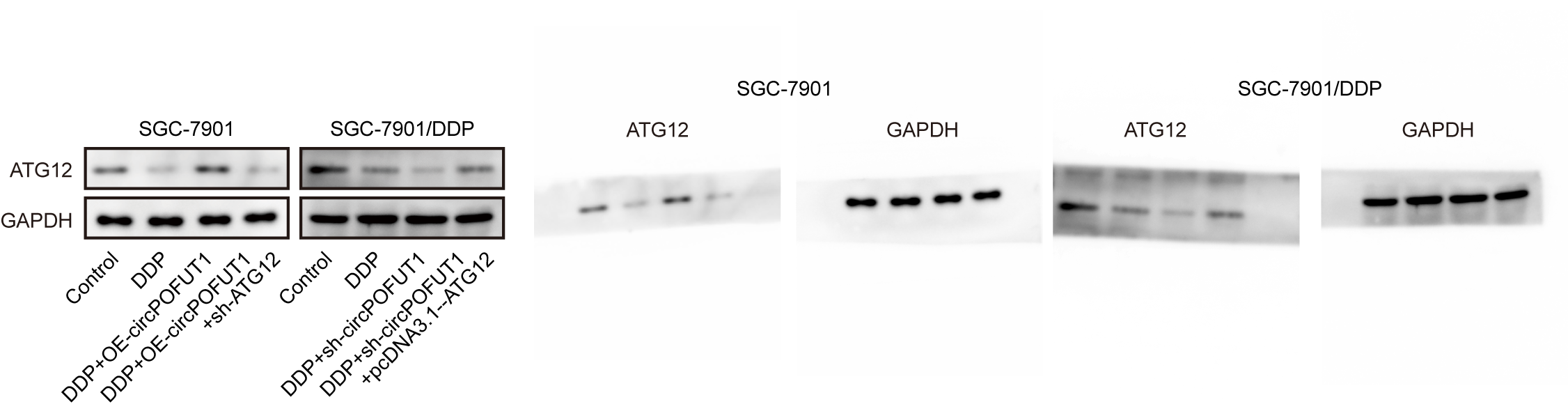


Fig7F


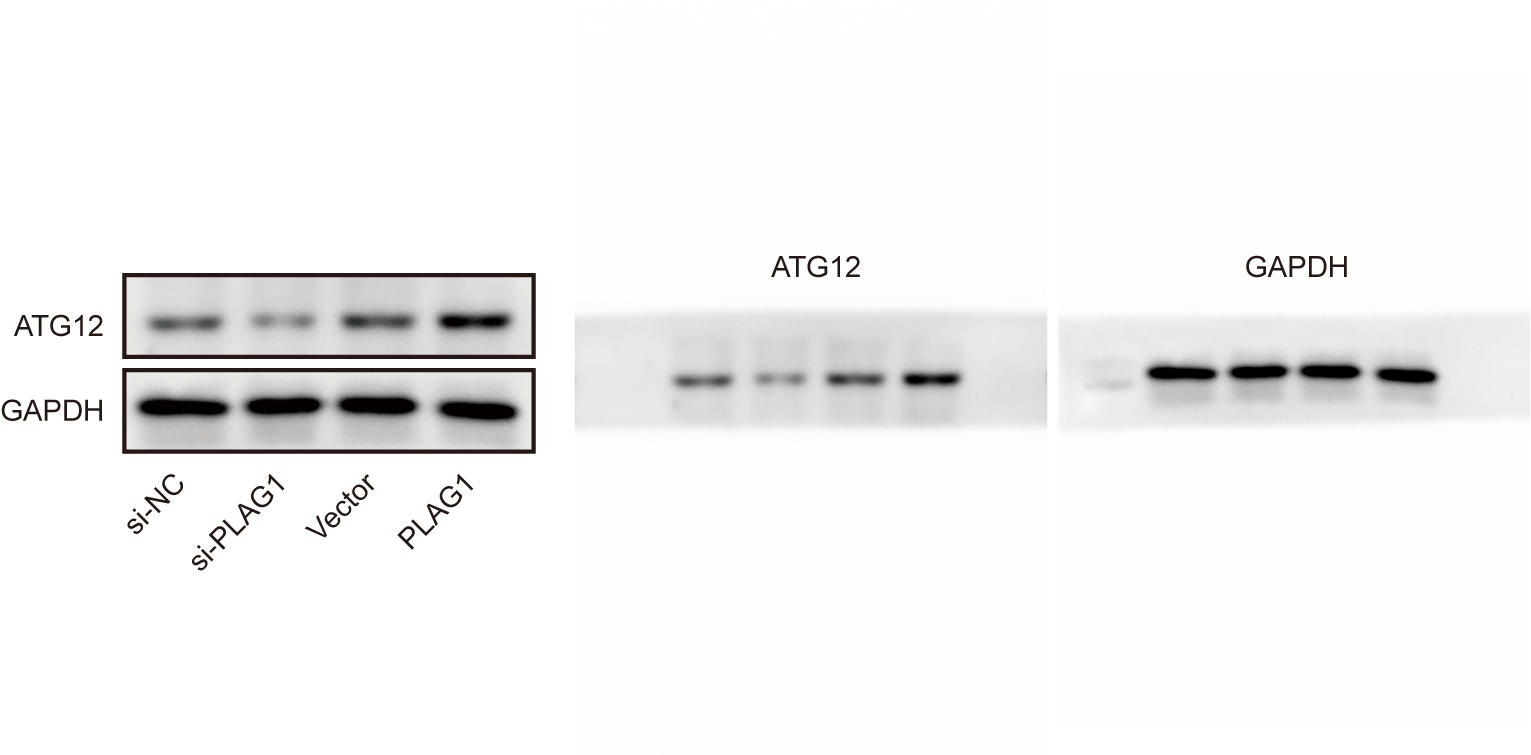


Fig8D


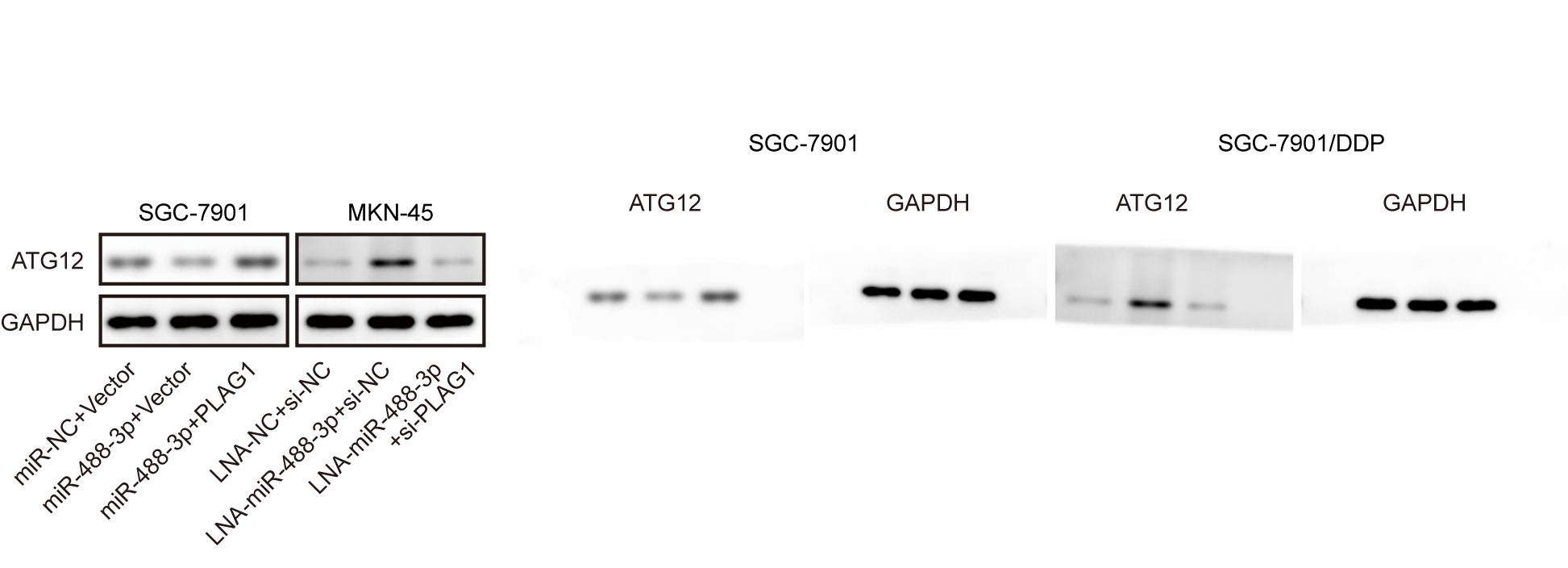

Supplement: Supplementary file 1 — Original Data File [file 41419_2022_5506_MOESM1_ESM.docx]
