# Supplementary material for: Circular RNA circPOFUT1 enhances malignant phenotypes and autophagy-associated chemoresistance via sequestrating miR-488-3p to activate the PLAG1-ATG12 axis in gastric cancer
Source: Cell Death Dis. 2023 Jan 9;14(1):10. doi: 10.1038/s41419-022-05506-0 (PMC9829716; doi:10.1038/s41419-022-05506-0)
Supplement: Supplementary file 2 — Supplementary Materials [file 41419_2022_5506_MOESM2_ESM.pdf]

Supplementary Materials

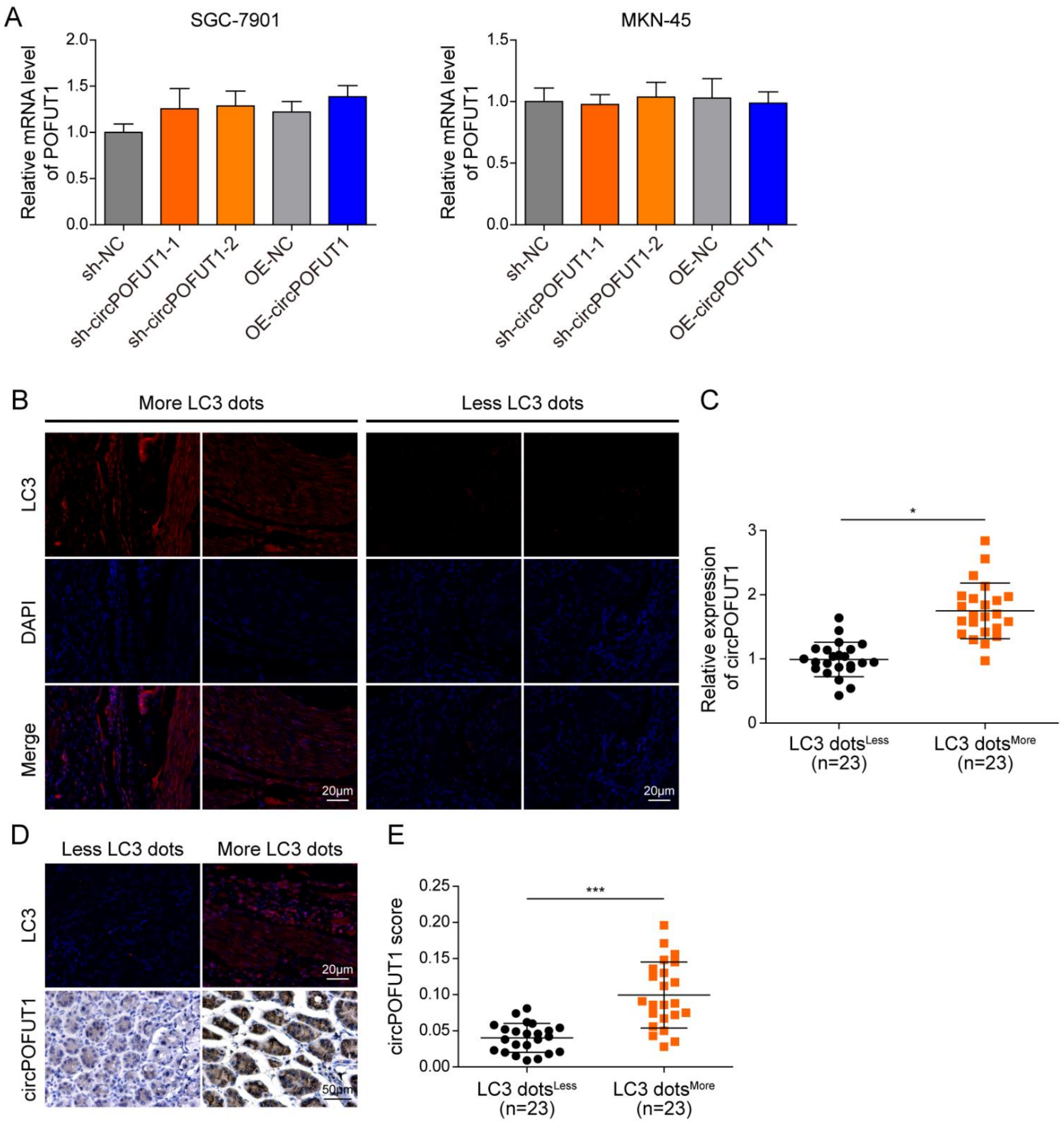

Supplementary Figure 1.

(A) RT-qPCR analysis of POFUT1 (n = 3).

(B)GC tissues were divided into LC3 dots ^{Less} and LC3 dots ^{More} groups according to the median number of LC3 dots (Scar bar = 20 μm)*.

(C) RT-qPCR was used to determine the expression of circPOFUT1 in GC tissues with LC3 dots ^{less} and LC3 dots ^{more} .

(D-E)In situ hybridization was used to determine the expression of circPIFUT1 in GC tissues with LC3 dots ^{less} and LC3 dots ^{more} (Scar bar = 50 μm).

*$P < 0.05$, *** $P < 0.001$.

A

B

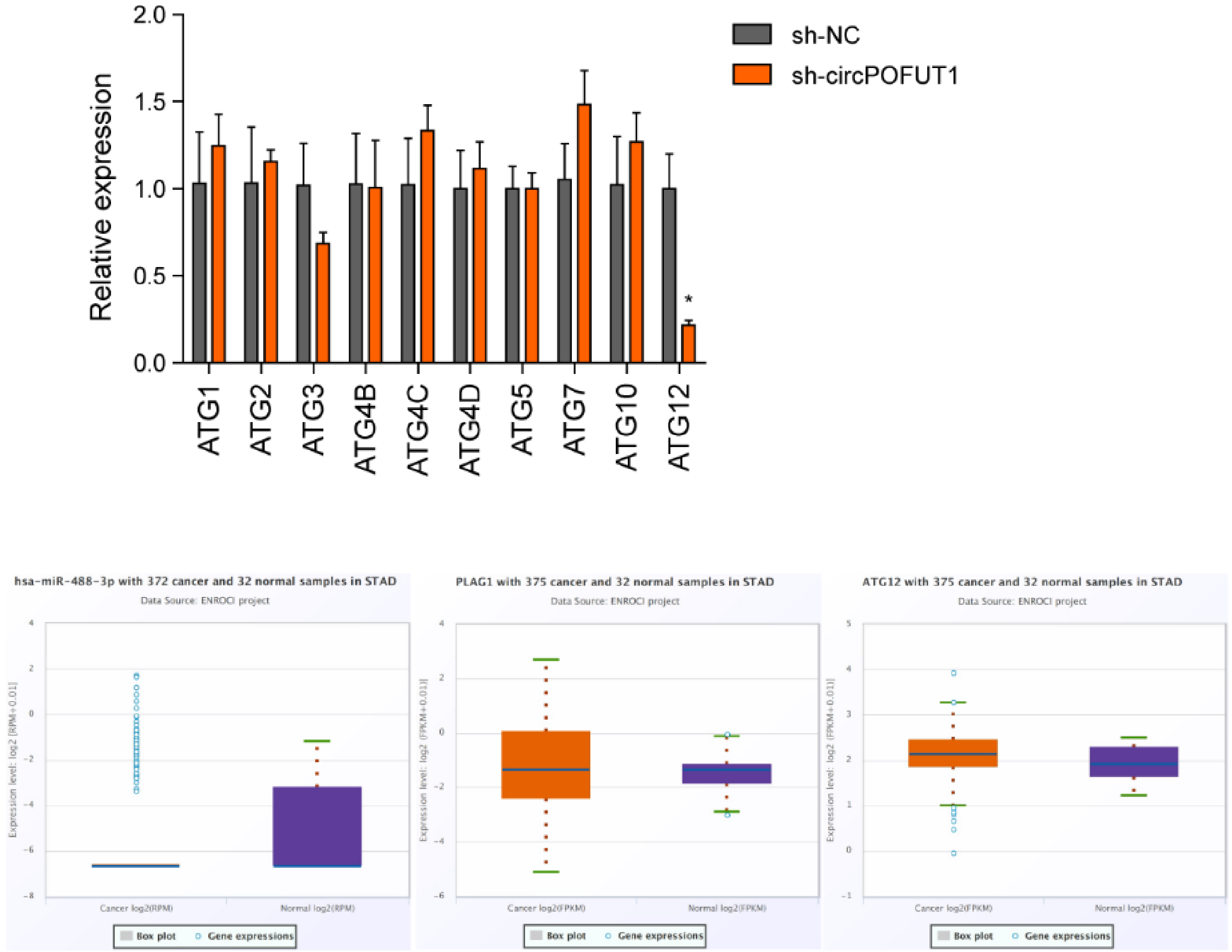

Supplementary Figure 2.

(A) The expression of ATG1-3, 4B, 4C, 4D, 5, 7, 10 and 12 in GC cells was analyzed by RT-qPCR (n = 3).

(B) Expression analysis of miR-488-3p, PLAG1 and ATG12 through TCGA.

*$P < 0.05$.

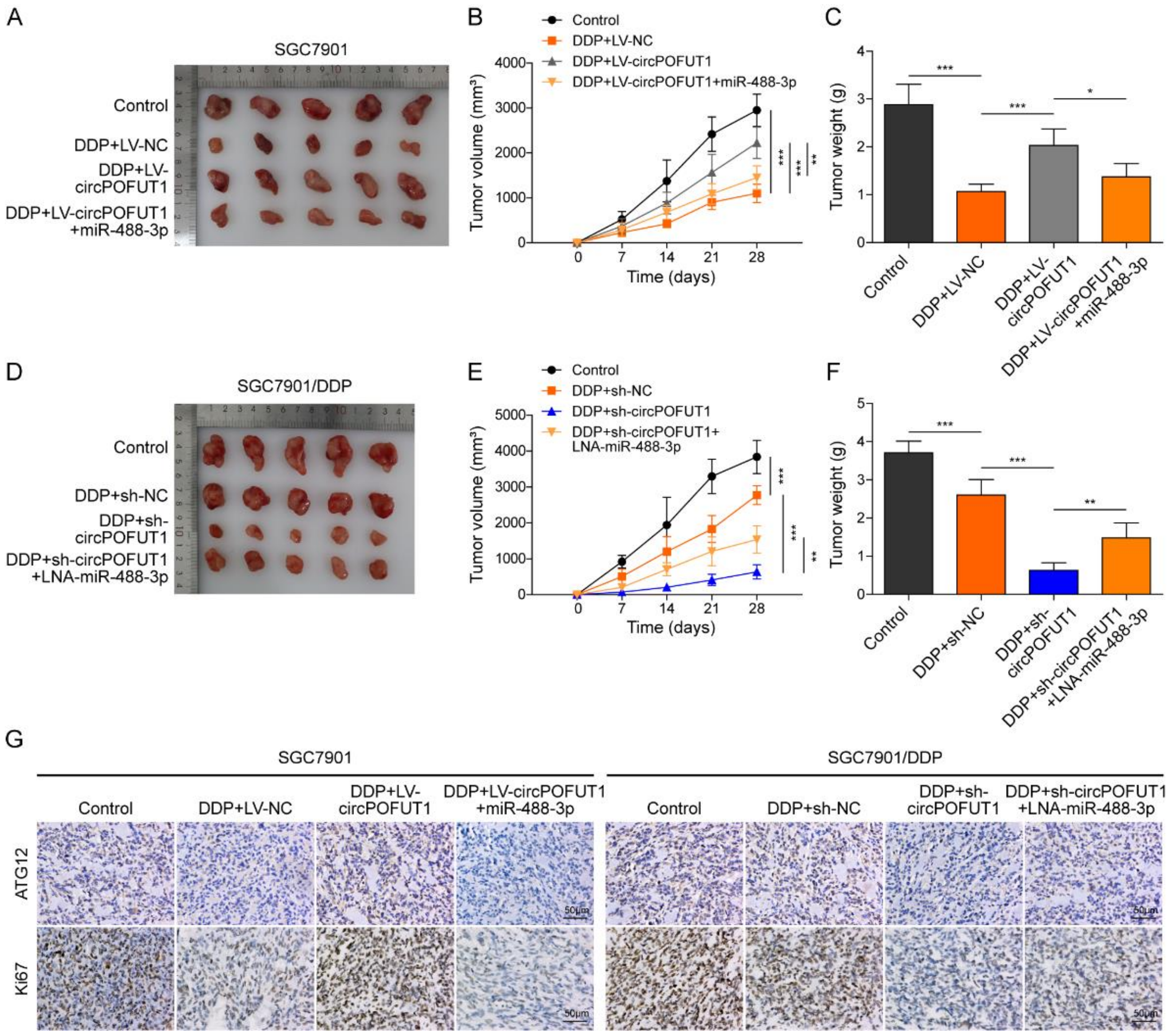

Supplementary Figure 3.

CircPOFUT1 accelerates tumor growth via targeting miR-488-3p to promote autophagy-associated chemoresistance *in vivo*.

(A) Photos of tumors (n = 5 per group) formed by SGC-7901 cells.

(B and C) The volume and weight of tumors formed by SGC-7901 cells (n = 5 per group).

(D) Photos of tumors formed by SGC-7901/DDP cells.

(E and F) The volume and weight of tumors formed by SGC-7901/DDP cells (n = 5 per group).

(G) IHC staining of ATG12 and Ki-67 (Scale bar, 50 μm).

*P < 0.05, **P < 0.01 and ***P < 0.001.

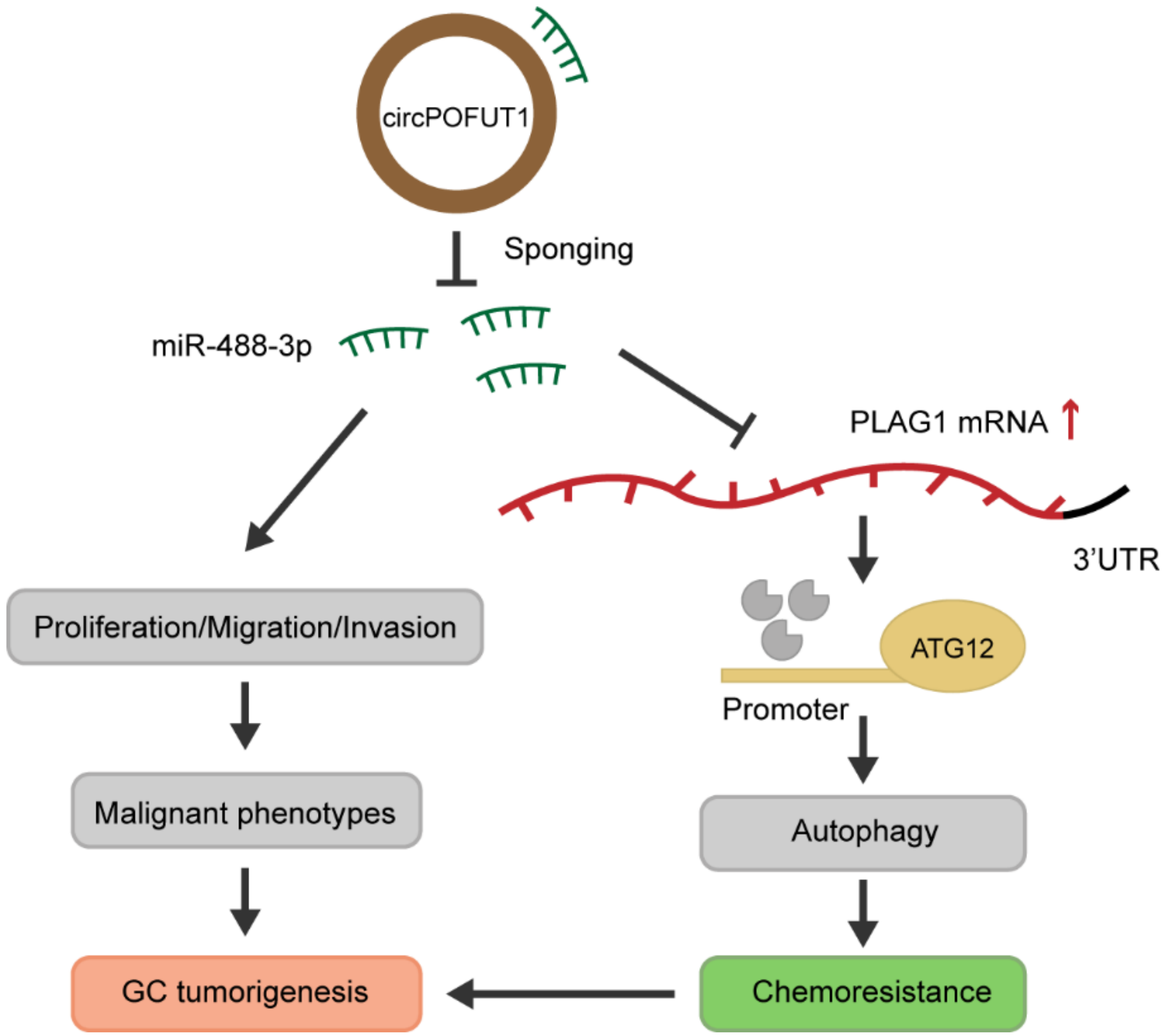

Supplementary Figure 4.

The schematic diagram of circPOFUT1 in GC.
